# Ambivalence towards discourse of disaster resilience

**DOI:** 10.1111/disa.12385

**Published:** 2019-07-19

**Authors:** Hanna A. Ruszczyk

**Affiliations:** ^1^ Assistant Professor (Research), Department of Geography and Institute of Hazard, Risk and Resilience Durham University United Kingdom

**Keywords:** community, disaster, disaster risk reduction (DRR), policy and practice, resilience, urban

## Abstract

This paper investigates empirically how the international aid community (IAC)—donors and practitioners—considers and implements disaster resilience in a specific country setting, Nepal, and throughout the rest of the world. A key finding is that there is ambivalence about a concept that has become a discourse. On a global level, the IAC utilises the discourse of resilience in a cautiously positive manner as a bridging concept. On a national level, it is being used to influence the Government of Nepal, as well as serving as an operational tool of donors. The mythical resilient urban community is fashioned in the IAC's imaginary; understanding how people create communities and what type of linkages with government urban residents desire to develop their resilience strategies is missing, though, from the discussion. Disaster resilience can be viewed as another grand plan to enhance the lives of people. Yet, regrettably, an explicit focus on individuals and their communities is lost in the process.

## Introduction

*It [resilience] helped to get better interdisciplinary discussion going*.

—Senior official with a multilateral donor organisation, 2015

*I hate the word resilience! This might be a good place to start. It is framed always in an academic context, and I see myself as a practitioner. … For me, resilience is the ability to survive and have a good life at the end of day. I think we could overly intellectualise it*.

—International disaster risk reduction expert, 2016

The two quotations above highlight the ambivalence that the concept of (disaster) resilience elicits within the international aid community (IAC). In the first case, a senior official working at the interface of disasters, climate change, and conflict in many countries states that resilience has allowed different stakeholders to engage in conversation, but he/she is reticent to suggest that it has changed his/her organisation. In the second case, an international disaster risk reduction (DRR) expert coordinating community‐based DRR (CBDRR) activities between international non‐governmental organisations (INGOs) and the Government of Nepal articulates the frustration of practitioners in utilising the concept of disaster resilience in the country.

Matyas and Pelling (2015, p. S5) point out that there is quite limited ‘empirical evidence on how resilience understanding is adopted and applied by practitioners, managers, community leaders and policymakers in disaster risk management’. Anderson (2015, p. 65) adds that ‘[w]e do not know what resilience is and we do not know what resilience does’ in practice. This paper attempts to fill this void with respect to the IAC (donors and practitioner INGOs). In particular, it seeks to illustrate how the IAC in Nepal uses the discourse of disaster resilience and what is lost through its employment.

The paper argues that on a global level, the IAC is considering the discourse of resilience in a cautiously positive manner as a concept that bridges the lacuna between different disciplines, such as climate change adaptation, development, and DRR. On a national level, disaster resilience is being used to influence the Government of Nepal, as well as serving as an operational tool of donors. Furthermore, the paper explores to what extent this framing includes residents and communities in urban settings in Nepal. Lastly, analysis of this grand plan of resilience leads to an assessment of what is lost in this discourse of disaster resilience: an explicit focus on people, power, and politics.

## Conceptual framing

### Resilience as a discourse

There is no agreed definition of resilience, nor should there be. Owing to its application within numerous disciplines, an agreed definition is not possible (Asprone and Manfredi, 2014). Resilience is widely seen as a desirable system property in environmental management (Klein, Nicholls, and Thomalla, 2003), giving it traction beyond the ecological field in complex human‐related spheres. Resilience, if viewed holistically, can bring together different perspectives (economic, environmental, human, physical, and social).

What is particularly relevant at this time is how the concept of resilience is being used and what this allows to happen in practice. Some academics view resilience as a form of neo‐liberal governmentality (Evans and Reid, 2013; Joseph, 2013; Chandler, 2014), whereas others continue to acquire inspiration from it (Brown, 2014, 2016). Kelly and Kelly (2016, p. 2) even contend, based on their research on the use of the concept by practitioners in the United Kingdom, that it is possible that ‘reclaiming resilience, building solidarity, and political agency can also go together’.

Resilience has become one of the leading ideas of our time to deal with uncertainty, change, and varied disruptions, as witnessed by policy discourse and academic debate on the matter (Hutter et al., 2013; Anderson, 2015). *Discourse*, as used here, follows the definition of Brown (2016, p. 38): ‘A common understanding of a phenomenon shared by a particular group of people. … A discourse may become institutionalised and may be important in shaping activity or directing policy’. For instance, in *Resilience Scan* #x007C; *July–September*, Kirbyshire et al. (2017, p. 2) explain that ‘as the “resilience revolution” in international development continues, researchers at ODI [Overseas Development Institute] are capturing the new direction and reviewing the latest thinking in this field’. Resilience has become a *field*.

Resilience has also become the policy ‘buzzword’ of choice for a range of international decision‐makers and it is impacting on traditional conceptions of governance. Resilience thinking influences how problems are perceived and addressed and the type of knowledge valued by decision‐makers (Chandler, 2014). Chandler (2014) asserts that resilience is a concept that has become central to government policy understanding. In the past two decades, for example, the *Hyogo Framework for Action 2005–2015* (United Nations International Strategy for Disaster Reduction, 2005) and the more recent *Sendai Framework for Disaster Risk Reduction 2015–2030* (United Nations Office for Disaster Risk Reduction, 2015) have advanced the disaster resilience agenda throughout the world.

Mitchell and Harris (2012) contend that the concept has been appropriated by bilateral and multilateral donor organisations. Resilience has become an ‘increasingly dominant mode of Western intervention in the global South’ (Pugh, 2014, p. 314). It is being discussed at the international policy level, position papers have been developed, and donor projects are being formulated to build disaster resilience, community resilience, urban disaster resilience, and other variations of resilience.

Apropos of disasters specifically, resilience has become ‘a seductive theory in disaster management’ (Lizarralde et al., 2015, p. S76). While this author does not agree that resilience is a theory, seductive it is to bilateral and multilateral donor organisations. Despite the concept's lack of rigorous empirical and theoretical grounding in the social sciences (Brown, 2012), it has significant purchase in the climate change, international development, and DRR fields (Brown, 2012). Moreover, resilience is being used to form a bridge between areas of policy and science, but there is limited evidence to suggest that the bridge is functioning properly (Brown, 2016). Resilience is ‘dangerous because it is removing the inherently power‐related connotation of vulnerability’, according to Cannon and Müller‐Mahn (2010, p. 623), who go on to claim that the ‘[r]esilience approach is in danger of a realignment towards interventions that subsumes politics and economics into a neutral realm of ecosystem management, and which depoliticizes the causal processes inherent in putting people at risk’ (Cannon and Müller‐Mahn, 2010, p. 633).

From a positive standpoint, the resilience discourse is bringing together ‘otherwise disparate groups, institutions, disciplines and scales’ (Tanner, Bahadur, and Moench, 2017, p. 3). In *Challenges for Resilience Policy and Practice*, Tanner, Bahadur, and Moench (2017, p. 3) also note that ‘[r]esilience narratives have been accused of a depoliticising effect by reframing issues in a way that makes populations affected by shocks and stresses responsible for securing themselves’. This paper provides conceptual and empirical evidence to support this statement.

### Operationalising resilience

Owing to exasperation among donors and practitioners worldwide at the fixation on definitions of resilience, practitioner literature has been concentrating on how to make resilience ‘useful’ or how to operationalise it, as a metric for project success. Operationalising resilience signifies the desire to develop indicators of resilience or benchmarks for assessing the concept in different contexts to make it a usable management tool for governments, policymakers, and practitioners globally.

Mochizuki et al. (2017) provide a literature review of community resilience measurement efforts. There are many unknowns with tremendous financial, political, and social implications for donors, practitioners, recipient national governments, and, most importantly, the people and ‘communities’ that are expected to be more resilient because of the external support received.

It is unclear if the operationalisation of resilience through the development of indicators can be or should be promoted further. Bahadur, Ibrahim, and Tanner (2013, p. 62) explain that one approach is ‘to develop a set of principles of measuring resilience rather than a universally applicable set of indicators’. Understanding the elements of resilience that present themselves after a disaster may help to shed light on how to build community resilience to natural hazards (Buckle, 2006; Solnit, 2009; Ride and Bretherton, 2011; Aldrich, 2012). However, the tensions associated with operationalising resilience are difficult to reconcile. Levine (2014, p. 2) suggests that the attempt to ‘find the perfect resilience index is not so much a difficult quest as a search for a holy grail’, distracting from more important issues such as how to improve the lives of millions of people across the planet.

Owing to the vast upsurge in the use of the term resilience, and the multitude of operational models developed and employed by practitioners, Schipper and Langston (2015) conducted a comparative overview of 17 resilience measurement frameworks, analysing indicators and approaches used by the IAC globally. They identified differing epistemic roots and definitions and concluded that there are limits to what indicators can provide and that ‘universal indicators cannot exist’ (Schipper and Langston, 2015, p. 9). They underscore that ‘the ability to measure resilience through consistent mechanisms is intended to enhance the accountability of funding for NGO [non‐governmental organisation] programmes, which is necessary for budgeting and public investment decisions, as well as offering a way of assessing progress towards resilience’ (Schipper and Langston, 2015, p. 9) in relation to project variables or the *Sendai Framework for Disaster Risk Reduction 2015–2030* or the Sustainable Development Goals (SDGs) set by the United Nations (UN) for 2030.

Schipper and Langston (2015) caution against practitioners becoming overwhelmed by the challenging and complex frameworks being developed and potentially losing the strategic view of their mission. For instance, in some of the frameworks that they reviewed, it is not clear whether or not indicators refer to ‘individual or group resilience’ or ‘who they are focused on, as in whose resilience is to be built’ (Schipper and Langston, 2015, p. 19). They conclude their comparative overview by stating that it would be useful for practitioners and donors to find some common ground Schipper and Langston (2015, p. 21):

*To ensure that rather than tearing each other down because we don't agree on how the concept is used, we can actually use this energy to help reduce the risk posed by climate change and natural hazards*.

This is a damning summary of the state of resilience thinking among donors and practitioners and of efforts to operationalise the concept. Béné et al. (2017, p. 212) emphasise that existing resilience measurement frameworks are ‘poorly adapted to the reality faced by practitioners on the ground’. There is clear frustration with efforts to operationalise resilience. While the drive to operationalise resilience may be of some value to donors and practitioners, it does not appear to offer much in the way of significance to people who live with and manage risk in daily life or when a disaster unfolds. Nightingale (2015, p. 194) notes that in Nepal, there is concern that the IAC is ‘devolving responsibility for resilience to locally based populations, and yet how they propose to do this, and what support is required to achieve these goals, is very different’ to what local people themselves think and express.

### Community, community resilience, and power

A brief interrogation of the concept of community is warranted at this point. In an exploration of etymological dictionaries, Esposito (2013, p. 15) states that community is derived from *cum* (with) *munus* (a task or duty). That is, a group of people who are together with a common focus. He argues that we ‘need communities’; they are ‘both necessary and impossible’ (Esposito, 2013, p. 15). Viewed from this perspective, communities are aspirational rather than a tool with which to achieve something else. If Esposito (2013, p. 20) is correct—the ‘only way to realize community would be to overcome interests and individual differences, but interests and differences are in fact insurmountable, because they are also what constitutes our nature‘—one needs to tread carefully in utilising the concept.

In the realm of disaster studies, Cannon et al. (2014) assert that ‘community’ is a myth, whereas de Beer (2013) considers it to be a romantic idea of the IAC. Ride and Bretherton (2011, p. 3) point out that DRR and disaster researchers ‘tend to assume that the community is a pre‐existing entity, one that needs to be educated otherwise changed to mitigate future hazards, risks and vulnerabilities to natural disaster and their effects’. This fallacy continues to be perpetuated.

Community resilience and the flow of power through spatial levels requires consideration (Wilson, 2012, p. 1219):

*Community resilience, therefore, is often associated with the quest for multiple resiliences within a community pursued by highly varying stakeholder networks, some of which may be directly contradicting and undermining efforts by other groups in the community to achieve maximum resilience*.

Wilson's definition of community resilience is associated with multiple scales, influences, and power. Bankoff et al. (2015, p. 8) argue that, in relation to communities, ‘power relations are almost always present (in a wide variety of configurations), especially on grounds of gender, class, ethnicity, caste, patron–client relations or age‐group bonding’. These dynamics are often difficult to make visible, but they wield tremendous influence on the way in which individuals and their communities can present their resilience.

## Methodology and a description of Nepal

### Methodology

Through a series of interviews with donors, INGOs, Nepalese practitioners, representatives of the Government of Nepal, and residents of two large municipalities of Nepal, this paper employs a qualitative comparative approach (McFarlane, Silver, and Truelove, 2016) to investigate understandings of resilience. Research methods included semi‐structured interviews, focus‐group discussions, photography, and observations of daily life. A total of 125 interviews and six focus‐group discussions were conducted in Nepali with the support of a research assistant, or in English where appropriate.

The study adopted a multi‐scale perspective, appraising how different scales (Swyngedouw, 1997) affect each other and how power and influence flow between scales (household, community, local authority, national, and international). The research was carried out in an iterative manner over a three‐year period (November 2013–October 2016) and coincided with the phases referred to by the IAC: ‘before, during and after’ the high‐intensity earthquake of April–May 2015 that claimed the lives of almost 9,000 people (United Nations Office for the Coordination of Humanitarian Affairs, 2015). A one‐month research trip took place in November 2017 to learn about governance changes on a national and local level.

To comprehend how urban disaster community resilience projects were being structured, 20 semi‐structured interviews were organised with members of the IAC located in the city of Bharatpur in south‐central Nepal, as well as in the capital, Kathmandu, and internationally. Semi‐structured interviews were also held with 60 residents in the cities of Bharatpur and Dhangadhi in the far west of the country (with populations of approximately 300,000 and 150,000, respectively). These people were selected with local support to ensure a cross‐section of society. In addition, 45 semi‐structured interviews were conducted with local‐, district‐, and national‐level government officials, as well as with key municipal stakeholders, including business association members, community leaders, construction sector employees, health volunteers, nurses, and teachers. Information garnered from these interviews set the context for understanding urbanisation, risk perceptions, resilience strategies, governance structures, and changing urban relationships. The data were coded into emerging themes and analysed.

### A description of Nepal

Nepal has made significant development gains in some respects. In fact, the country is regarded as a success story in terms of the Millennium Development Goals (MDGs) of the UN. According to the *Millennium Development Goals Needs Assessment for Nepal 2010* (Government of Nepal, National Planning Commission, and the United Nations Development Programme, 2011), despite the decade‐long conflict (in which approximately 13,000 people died) and political instability, progress has been significant in a number of areas. This has continued with the SDGs (Government of Nepal, National Planning Commission, 2017).

The MDGs, such as education and mortality, highlight the significant advances that the Government of Nepal has made on behalf of its population (Government of Nepal, National Planning Commission, 2017). Poverty has decreased very rapidly in the country in recent decades (Government of Nepal, Central Bureau of Statistics, 2012, section 5, p. 9): 41.8 per cent of the population was living below the poverty line in 1995–96, whereas the proportion was 25 per cent in 2011—the rate was even lower in urban areas: 15.46 per cent (Government of Nepal, Central Bureau of Statistics, 2012, section 4.1, p. 4). By 2015, the rate had fallen to 21.6 per cent (Government of Nepal, National Planning Commission, 2017).

Unfortunately, Nepal ‘remains one of the world's 48 “least” developed countries even after more than sixty years of “development”’ (Rigg et al., 2016, p. 64). With regard to the economy, ‘almost half of all households in Nepal have either a current or returnee migrant’ (World Bank, 2011, p. 26), illustrating how difficult it is to earn a livelihood in the country. Its economy is extremely dependent on international mobility and the income of its young men (World Bank, 2016). Remittances comprised 14 per cent of gross domestic product (GDP) in 2000; 22 per cent of GDP in 2010; and 32 per cent of GDP in 2015. The national economic situation is sufficiently precarious to force an estimated five million Nepalis to leave their families and earn livelihoods abroad (United Nations Development Programme, 2017).

Three key events occurred during the period of this study:

- first, a 7.8 magnitude earthquake struck on 25 April 2015, followed by a series of aftershocks, most notably a 7.3 magnitude tremblor on 12 May 2015;
- second, the National Constituent Assembly promulgated a new constitution in September 2015 after deliberating for seven years; and
- third, the number of municipalities in Nepal increased by 275 per cent, creating a nominally more urban country.

The political changes led to local elections in 2017 (the first in 20 years) and the establishment of a new form of federalism, which is leading to significant restructuring of the public sector. In spring 2017, the new federal government accorded the locally elected authorities significant responsibility, but the financial and human resources needed to implement their new mandate are in a state of flux.

## What does the discourse of resilience allow?

### Globally, resilience discourse bridges a gap

Climate change, conflict, development, and DRR were addressed in a siloed manner until recently. The discourse of resilience bridges silos of thinking and creates a conceptual opening and a common meeting ground for different debates. Such conceptual space can facilitate exploration of how to ‘work across silos’ (Levine et al., 2012, p. 1) and how to develop interrelationships (Schipper and Pelling, 2006; Bahadur, Ibrahim, and Tanner, 2010; Béné, Newsham, and Davies, 2013; Matyas and Pelling, 2015). It is a unifying concept (Mitchell and Harris, 2012) that minimises dichotomous thinking and spotlights linkages between the natural and social spheres (Rival, 2009). The necessity of this space for conversation and dialogue should not be underestimated.

The senior official responsible for climate change, disasters, and conflict on a global scale whose quote opens this paper explained that the concept of resilience ‘helped to get better interdisciplinary discussion going. There are now more diverse actors talking together in the same room’ (including climate change, development, DRR, and humanitarian specialists). He/she went on to note:

*Resilience is definitely a buzzword in my opinion. … It is a good thing, the idea behind it. It took root. A lot of organisations are working to embed it in the work they do, and trying to work differently. So it [resilience] will stay. Maybe not in the way it was talked about… years ago [operationalising it], but as an approach in terms of the way we need to change the way we work*.

Resilience is being viewed in a cautiously positive manner, as a new way of thinking among the IAC, allowing for a space to be created where new forms of collaboration within the IAC can be initiated and implemented. In the same meeting, a colleague of the senior official from a multilateral donor organisation added:

*In my view, it [resilience] is nothing new. … There is a need for the [international aid] community to come up every now and then with a new term [around which] to gather together. There will be something else that will be pushed up in a few years, but it does not mean that resilience will not stay*.

These two international representatives suggest that resilience is a mechanism through which donors and INGOs within the IAC can frame their work in a different manner. Resilience as a concept is useful to them, as it is allows donors and practitioners to consider their portfolio of work in a different way, but not to lose sight of the respective organisation's mandate. ‘Resilience reflects and seeks to offer a positive alternative to the loss of modern frameworks’ (Pugh, 2014, p. 314). Resilience as a bridging mechanism is positive and valued by these interviewees.

There will be disillusionment within the IAC if resilience is ‘pushed to represent more than it can deliver’; 'the problem lies in attempts to make resilience a full‐scale paradigm, which it is not' (Alexander, 2013, p. 2713). Conversely, on a global level, Joseph (2013, p. 50) argues that resilience may be now:

*Overused to the point of banality so that what was once referred to as putting down sand bags to stop flooding or ensuring that there are separate toilets for men and women are now described as resilience measures. The difficulty, therefore, is picking out usages of the term that have some genuine meaning … the key connection is governance. And again, this governance is working from a distance*.

### Resilience discourse influences the Government of Nepal

The IAC, comprising donors and INGOs, influences national and local government‐level priorities as well as emerging discussions concerning climate change adaptation, development, and DRR. Donor interventions and the actions of state government ‘interact with transnational imaginaries, contributing to the flow of meanings and shaping institutional spaces and practices’ (Berry and Gururani, 2015, p. 6). This form of governmentality is evident in Nepal, and is applicable to other development assistance recipient countries as well.

The IAC is powerful and its views hold sway over the Government of Nepal. Not only did it introduce DRR and disaster resilience discourse in Nepal, but also it defined debate on these matters. It wields considerable power in Nepal, owing, to a large extent, to its significant financial contribution to the country (Jones, Oven, and Wisner, 2016). As Jones, Oven, and Wisner (2016, p. 34) stress: ‘It is clear that the influence of international organisations in Nepal is very significant and that the donor community plays a large role in advancing the DRR agenda, especially earthquake risk reduction’. In Nepal, the IAC has supported the development of disaster management plans on a national, district, and municipal or village development committee (VDC) level. In addition, it has been working on CBDRR initiatives to develop disaster‐resilient communities under the framework of Flagship 4 of the Nepal Risk Reduction Consortium (NRRC)—the latter was disbanded in 2016 and Flagship 4 continued in a modified form in 2017.

Governments in donor countries are influencing national decisions in recipient nations such as Nepal, including which hazards and risks are most important and how to mitigate disasters. Joseph (2013) does not think people and the communities they create play much of a role in the resilience discourse described above. They are not the priority in relation to governing for resilience. Instead, he views resilience as a device ‘in an artificial construction where the real targets are states and governments’ that need to be managed by the IAC (Joseph, 2013, p. 51) in order to implement sectoral international priorities such as DRR.

This author agrees with him: the IAC is using resilience to steer national governments' limited human and technical resources towards DRR, in particular, rather than to produce a holistic understanding of risk. By so doing, some national and local factors that may enhance (disaster) resilience are ignored, such as strengthening linkages between government and people (Ruszczyk, 2017), strengthening livelihoods (Ruszczyk, 2014), and addressing a fuller continuum of risk (Ziervogel et al., 2017; Ruszczyk, 2018).

The recent past shows that ‘resilience thinking’ provides a mechanism for dialogue as well as possible collaboration and holistic thinking within the IAC. Where this begins to fray or fall apart is outside of the IAC. Matyas and Pelling (2015, p. S2) state that resilience is a new term but that there may be ‘no new action on the ground’. Pelling (2011, p. 51) also proposes caution in utilising the concept of resilience:

*The power of resilience to suppress deeper changes in the institutions and values that shape development and risk management is reinforced by its attractiveness as a solution … for donors and government precisely because it does not challenge the wider status quo*.

The discourse of resilience and resilience thinking is of limited use to national actors in Nepal. The discomfort and even hostility directed towards resilience as a framing mechanism can be seen in the perspective of a Nepalese disaster‐oriented NGO. A senior official with this organisation said the following about the drivers of resilience and DRR in Nepal:

*Who is leading DRR in this country? Who is the main driver of DRR in this country? Basically it is the foreigners! The government is basically guided by foreigners. For me, right from the very beginning, without the involvement of the local people and local culture and local authorities, I do not accept [the premise of] any of the DRR programmes. They are bound to fail!*

Jones, Oven, and Wisner (2016) found similar views about DRR and resilience in their comparison of the governance landscape of earthquake risk reduction in Nepal and the state of Bihar in east India.

### ‘Disaster community resilience’ is an operational tool

The concept of resilience is allowing conversations to take place on a global level, and it may be deconstructing siloes. While this is of significant international merit, on a national level, where donor projects are implemented, resilience may not have significant conceptual value. Rather, it is being used as an operational device to manage projects. Resilience is a discourse that has been instrumentalised to suit the needs of donors that have to account for their efforts in a more systematic manner.

In Nepal, disaster community resilience is an operational tool of donors vis‐à‐vis the INGOs working in the country. Donors and INGOs that, together with the Government of Nepal, were involved as part of Flagship 4 of the NRRC (Nepal Risk Reduction Consortium, 2013), developed nine minimum characteristics of a disaster‐resilient community, in an attempt to operationalise resilience. These nine minimum characteristics were incorporated, from 2012, in the disaster risk management projects and programmes of practitioners funded under Flagship 4—the latter's budget was USD 44.3 million between 2011 and 2016 (Oven et al., 2017).

The nine minimum characteristics of a disaster‐resilient community are:

- organisational base at the VDC/ward and community level;
- access to DRR information;
- multi‐hazard risk and capacity assessments;
- community preparedness/response teams;
- DRR/management plan at VDC/municipality level;
- DRR funds;
- access to community‐managed DRR resources;
- local‐level risk/vulnerability reduction measures; and
- community‐based early warning systems.

An IAC respondent working on the nine minimum characteristics (at the interface between donors, practitioners, and the Government of Nepal) underlined that the initiative:

*Has been encouraged, heavily encouraged, by donors along with impact analysis and assessment because there is pressure from donors who are getting pressure from their governments, who are getting pressure from constituents, about where all this money goes*.

In response to the question ‘why is resilience being used in this manner?‘, the same person said:

*But what else are they [donors and INGOs] going to use? Resilience is the term now that everyone understands and is so generalised that you can apply it to any field. … What other term is there for health, education, disaster, climate change?*

The reality is that ‘resilience’ does not have a common definition and decision‐makers who apply the concept in Nepal (the IAC and the national government) do not have a common understanding of its meaning. The minimum characteristics of a disaster‐resilient community may not be particularly relevant to people and communities according to the IAC respondent cited above. The nine baseline components do not address fully the range of risks and hazards as perceived by people. According to this particular IAC respondent, the minimum characteristics are responsible for:

*Creating a collective and creating a mass movement of INGOs, people with money [donors] and of the government [of Nepal]. It is both. The nine characteristics are a marketing tool. That is what they are. They are a marketing tool to get the attention of and to try to make the concept of disaster management more understandable [to the government]*.

He/she added:

*I do not think they [the minimum characteristics] are resilience … they are just a way to package it, but not a definition of resilience*.

The situation in Nepal reflects the state of affairs around the world. The framing of resilience has altered from ‘building back better’ (Monday, 2002, p. 1), to ‘bouncing back’ (Twigg, 2007), to ‘bouncing forward’ (Manyena, 2009) to a better future. In a seminal article, Manyena (2006, p. 436) contended that disaster resilience could become a new phrase, the primary value of which would be its description of ‘a desired outcome of a disaster risk reduction programme’. Levine (2014) claims that the desire to operationalise or quantify resilience is an attempt by donors and practitioners to account for funds granted. This appears to have become a reality in Nepal and internationally.

## Problematising resilient communities

### The mythical resilient (urban) community

The Ministry of Federal Affairs and Local Development (MoFALD) states that responsibility in the event of a disaster will lie primarily at the community (neighbourhood) level. A senior government official in the ministry, which ceased to exist in 2018, replaced principally by the newly created Ministry of Local Development and General Administration (MoLDGA), proposed the following definition of a disaster‐resilient community:

*The community is central. Government and other partners can improve their capacity to deal with a disaster. Government is a small part in the capacity of the community. Communities that have sufficient capacity to save their lives and property from disaster, we think if these communities have these capacity, these communities are resilient*.

This is a particularly narrow interpretation of a disaster‐resilient community, with minimal thought paid to complexity, inter‐linkages, scales, and the role of government and other partners. The focus is on recouping losses; there is little consideration of the future.

The IAC worked with MoFALD for several years to support CBDRR initiatives. Many of the projects are striving to develop disaster‐resilient communities under Flagship 4 of the NRRC. The senior government official cited above stressed that the community is at the heart of disaster resilience at the local level. The urban community needs to take care of itself; the government does not have a large role to play in helping communities become resilient. It is unclear how he/she expects communities to help themselves and using whose resources. The national government is abdicating responsibility to its people.

A decades‐long myth continues to be perpetuated that communities are ‘capable of anything … all that is required is sufficient mobilisation (through institutions) and the latent capacities of the community will be unleashed … the evidence does little to support such claims’ (Cleaver, 2001, p. 46). The MoFALD representative argued that government should fulfil a support function; it only needs to play a small role. The lowest level of formal government in Nepal is the ward; there is no formal public sector mechanism to support the neighbourhood level. Consequently, the government has the flexibility to decide whom to support in a time of crisis or disaster and whom to ignore owing to informality of government procedures (Ruszczyk, 2017).

The senior official problematised communities in an urban municipality: ‘In one ward there will be four or five communities, [it is] difficult to merge these four [to] five communities into one, and each community has its separate problems’. He/she pointed out that urban communities, smaller than the ward geographically, are expected to be self‐reliant with minimal support from government because the latter does not have the resources and the capacity.

The official, based in Kathmandu, clearly understands the tension between how the national government, with the financial and technical backing of the IAC, is structuring disaster resilience and the role of the public sector in managing resilience‐building efforts. For instance, MoFALD, and its successor, MoLDGA, require that municipalities have disaster management plans, including at the ward level. The central government explains that local authorities will have a budgetary provision for disaster resilience. Local governments have newly elected ward presidents but there is no formal mechanism to establish a connection with the government's interpretation of a community—that is, the neighbourhood group.

### People and their communities are missing

The phrase ‘disaster community resilience’ is useful for the IAC. Through the discourse of resilience, donors and INGOs can work under an operational framework that structures their work in a new programmatic manner. The need to work in a different way has been an issue for decades, but the resilience discourse is creating a testing ground for new means of implementation. Regrettably, however, an explicit focus on people is absent from this discourse. Unacknowledged are the ‘beneficiaries’ of these IAC projects; the communities of people who are expected to be resilient—in relation to what and over what time period is unclear.

There is not much of an overlap between communities targeted by projects and organically created forms of community or networks that exist already. There is a disconnect between the manner in which the IAC is working in Nepal and how urban communities organise and attempt to address their perceived continuum of hazards and risks. An IAC respondent who has worked for many years on the nine minimum characteristics in Nepal asserted that donors and INGOs view ‘community as just this other thing at the end, which they then try to shove into a box for measurement purposes’.

Communities formed organically by residents fall away as an object of analysis in this disaster‐resilience discourse of the IAC. In this operational framework of quantification, log frames and accountability with regard to funding, individuals, and communities are missing. The rush to redistribute responsibility for disaster resilience from government to communities is increasingly problematic if the role of government is lost in the discussion and the burden for being resilient is on the individual or on urban ‘communities’.

There are indications, based on the author's interviews in late 2017 with recently elected local government officials, that local authorities intend to work with all (informal) neighbourhood groups. Challenges, though, include the fact that in many cities, there are no neighbourhood groups or residents are excluded from neighbourhood groups owing to caste/ethnicity, socioeconomic class, or landownership status. These urban residents will struggle to be resilient without the support of these key social structures in the cities.

If the focus is on community in the form of neighbourhood groups, the more vulnerable members of society are left unseen, unheard, and unable to become resilient to unforeseen disasters. In the past, the development ‘gaze turned peasants, women, and the environment into spectacles’ (Escobar, 2012, p. 155). One could argue now that the international DRR and development discourse is establishing so‐called resilient communities as the spectacle without genuinely engaging with people and communities. Understanding how people create communities and what type of linkages to government they desire in building their resilience strategies (Katz, 2004) is missing from discussions nationally and internationally.

By employing the phrase disaster‐resilient community vis‐à‐vis CBDRR projects, the IAC in Nepal is generating an illusion of supporting the resilience of people to natural hazards in Nepal. This criticism (of not engaging fully with people) is not peculiar to resilience; it is in fact a feature of many development interventions in the country and abroad. Nevertheless, it still warrants interrogation in this analysis of disaster resilience.

## Analysis of a grand plan

### Disaster resilience and the complexity of the everyday

Disaster resilience can be considered as another grand plan (Scott, 1998) introduced by the international community to enhance the lives of people in the Global South. The concept of disaster community resilience allows outsiders, such as the IAC in Nepal, interested in influencing the structures and behaviour of governments and groups working on DRR to stake a claim concerning the impacts. Yet, disaster community resilience does not engage sufficiently with urban residents, their continuum of urban disaster risk, their sources of and access to power, and the politics involved.

As Joseph (2013, p. 52) underscores: ‘The limits to resilience are real. Although it might increasingly pervade international organisations, this does not necessarily have any meaningful effects on the ground’. Resilience has been introduced with well‐wished desires (positive change for people) that prove difficult to achieve in practice. How the concept is being used within the disaster resilience discourse, and the approach supported by donors in Nepal, are at odds with the needs of people in cities.

There is increasing concern among the IAC about the application of the concept of resilience. Resilience has been encapsulated in a wider debate critiquing development. In addition, there is awareness that in Nepal, as in other places, ‘development’ has helped to manufacture risk (Cannon and Müller‐Mahn, 2010). The relationship between development and disasters necessitates consideration at this point. Disasters should be understood as unsolved development problems since they are not events of nature, but, rather, the product of the interaction of people, society, and the natural environment (Cardona, 2004).

In a paper entitled ‘Engendering development and disasters’, Bradshaw (2014) calls for an explicit focus on the root causes of women's vulnerability, and suggests that IAC programmes be designed to concentrate on reducing gender inequalities by challenging unequal power relations. With regard to this paper, it is contended that donor projects should focus explicitly not just on gender, but also on people, urban residents, the root causes of vulnerability (Wisner et al., 2004), and the continuum of perceived risk that individuals face in the everyday, including to their livelihoods and economic security (Ruszczyk, 2014, 2017, and 2018). An international DRR expert in Nepal made the following observation:

*What I really think and from what I have seen, in order to talk about resilience, you have to talk about the bigger context of how to improve livelihoods. … So you cannot talk about [disaster] resilience if people have nothing. And they have no capacity to think about tomorrow*.

This respondent argues for a more holistic understanding of development, differentiating between disaster resilience to a hazard and resilience as a more general concept, whereby people have opportunities for a better life on their own terms. Increasingly, members of the IAC are dissatisfied with the narrow application of resilience to a hazard. Instead, they are seeking a more holistic understanding of the risks and hazards that confront residents. The IAC is critiquing ‘development’ in Nepal. The DRR expert cited above suggests that international policymakers and Nepalese people comprehend issues in very different ways.

In an interview after the earthquake in 2015, another international expert working on CBDRR highlighted interrelated issues (exclusion based on caste and the relationship between the economy and governance) that CBDRR resilience projects have ignored until now. He/she stated that, within Nepalese society:

*Exclusion is based on caste basically. There is a lot of corruption. The [national] budgets are not used properly. The budgets are used to reinforce the system. Basically, when we talk about resilience, when we talk about systems, in Nepal the system is actually so broken down, the communities are on their own for most of it. So [all of us] need to address [the] governance issue. This would be the starting point. Also poverty and [the] economy. The livelihoods of the people do not give them enough space to get out of the poverty trap. [The focus of the IAC] should be less about disasters and more about other things in my opinion. More about [the] economy and governance. Disasters are important, but the individuals are working all year to get out of [the] poverty trap, the disaster reduces a bit of effectiveness or efficiency of what they have achieved*.

He/she explained that historically, the government has been seen as an exploitative structure against large portions of the Nepalese people, adding: ‘I think they [the Nepalese people] are [being] pushed to the limit’. Nepalese people cannot do more for themselves. In reality, they are already resilient subjects (O'Malley, 2010; Evans and Reid, 2013). They need other governance scales to support them, such as the newly elected local authorities and the new federal government (as of 2017), in order to have a better quality of life and a safe future.

### What is lost by using the discourse of resilience?

People, power, and politics are lost in this grand plan of (disaster) resilience. An understanding of the priorities of people and how they already cope and show their resilience is absent. People's expressed desire for safety is lost in the discourse of resilience. Bahadur and Tanner (2014) underscore the need to consider people, power, and politics in their exploration of how the concept is employed within the Asian Cities Climate Change Resilience Network initiative in India.

Donors and practitioners bring their own priorities (DRR and an emphasis on particular hazards), language, and tools (such as CBDRR and disaster resilience) to Nepal and other countries that receive funding. Resilience discussions in the realm of climate change and disasters are based, to a significant extent, on Western concepts and thus are not particularly relevant in the Global South (Voss and Funk, 2015). The language of resilience allows international and national policymakers to hide behind people who are accorded responsibility for helping themselves in times of hardship. These are people who are largely ‘pushed to the limit’ in the words of the international CBDRR expert quoted earlier. Focusing on disaster resilience is insufficient in this context.

Why and how resilience is of value or a necessity, as well as by whom and for whom, are matters that are also not often addressed. Carpenter et al. (2001) were the first to ask ‘resilience of what to what?’, a question that remains pertinent. Drawing on the understandings of development and science and technology studies, Leach (2008, p. 3) suggests querying: ‘resilience of what, for whom?’. If urban residents, communities, and cities (Vale, 2014) are to enhance their resilience (or be more than resilient) to an event, a natural hazard (Tobin, 1999; Gaillard, 2007; Fernando, 2012) or specifically a seismic hazard (Bruneau et al., 2003; Ainuddin and Routray, 2012), then power issues need to be considered. Slater (2014, n.p.) even hypothesises that ‘“resilience” studiously, perhaps even judiciously, ignores every important question’ in relation to uneven development, enabling, inter alia, political structures and state strategies.

Only by asking questions such as ‘whose resilience is important?’ to ‘what event or hazard?’, ‘whose lens is being used to examine resilience?’, ‘who impacts on resilience?’, and listening to and comprehending the power relations, the range of scales involved, and the complex intersectionality between those scales, can resilience as a concept be used to benefit those who need to be more than resilient subjects. It is here that further exploration of people's perceptions of risk, economic security, social networks and communities, and relationship to government is warranted, not only in Nepal but also in many other countries.

## Conclusion

This paper investigates empirically how the IAC (donors and practitioner INGOs) considers and implements (disaster) resilience internationally and nationally in Nepal. The country setting of Nepal is specific (a post‐conflict and hazard‐prone nation), but the general themes of this analysis can be generalised to other parts of the world where the IAC has introduced the (disaster) resilience discourse.

The two quotations at the beginning of the introduction suggest that the discourse of resilience can be viewed positively and negatively within the IAC. On a global level, the IAC is considering the discourse of resilience in a cautiously positive manner as a bridging mechanism via which donors and INGOs can attempt to work together more effectively across the areas of climate change adaptation, conflict, development, and DRR. On a national level, disaster resilience is being used to influence the Government of Nepal, as well as serving as an operational tool of donors. Disaster community resilience is a project management instrument of donors to measure the effectiveness of CBDRR projects in Nepal.

Regrettably, however, an explicit focus on people and their communities is lost in the process. The mythical resilient urban community is fashioned in the IAC's imaginary; understanding how people create communities and what type of linkages with government urban residents desire to develop their resilience strategies is missing, though, from this discussion. The bottleneck in supporting urban residents to be more resilient is to be found within both the IAC and the national government.

Disaster resilience can be viewed as another grand plan introduced by the international community to enhance the lives of people. The concept allows the IAC in Nepal to influence the structures and behaviour of government and practitioners working on DRR in order to stake a claim to the impacts. However, disaster community resilience does not engage sufficiently with urban residents, their understanding of urban risks and hazards, their sources of resilience, and the ways in which people interact with the government to build a safe future.

If the IAC community could listen to residents' perceptions of risk and understand the social networks already in place, its interventions would be structured in a different manner. This would entail more of a spotlight on livelihoods, urban infrastructure, and the relationship between government and people, and would be a starting point in shaping a safer future. Governance would play a key role here; linkages between people and government would be enhanced so that they can work together in the short and long term on everyday and infrequent risks, including hazards such as earthquakes and floods.

These types of projects may be difficult to account for in a log frame or within other project management tools. The benefits of such projects would also not be immediate, making it difficult for INGOs to demonstrate the impacts of their projects to donors. A focus on people, power, and politics has been lost through the discourse of resilience and urban residents are left behind to take care of themselves and to create a safe future. People deserve more—the effort needs to be made.

## Acknowledgements

This paper is dedicated to Dr Bernard Manyena, who died on 27 April 2018. His paper entitled ‘The concept of resilience revisited’, which appeared in *Disasters* 30(4), introduced me to the concept of resilience. He was a thoughtful scholar who was particularly interested in policy and practice.
